# Spirit of the British and American Periodicals

**Published:** 1837-10-01

**Authors:** 


### Spirit of the British and American Periodicals.

### The Present State of Pharmacy in Germany.*

Mr. Kane, the scientific chemist, of Dublin, has communicated the paper from which we extract the following particulars to our Irish contemporary.

It would appear from Mr. Kane's observations, that, if England is the land of *pure* surgeons and physicians, Germany rejoices in her *pure* apothecaries.

"In attempting, by the following remarks, to dissipate those incorrect ideas, I shall confine them to the state of pharmacy in Germany, as in that country the pure apothecary exists in a degree of purity unknown elsewhere, the laws in France, as we shall cursorily remark, reducing the profession to a very inefficient condition. And it is fortunate for the simplicity, as well as for the brevity of the communication, that the differences between the regulations of the various German states are so trivial, that a description can be found almost equally applicable to all."

The following is the situation of the German apothecary.

The grand distinction between the apothecary in Ireland and in Germany, is, that the latter is, in fact, an officer of the government. On his being pronounced by competent examiners properly qualified for the office, he is, on the occurrence of a vacancy, appointed to dispense medicines to the sick people; and the government, in place of paying him a direct salary from the public purse, enables him to pay himself, by charging for his medicines, the price being fixed by authority, and competition being prevented, inasmuch as none but apothecaries are allowed to retail drugs; and the number of apothecaries is kept within a certain limit.

We fancy that the latter regulation would be no bad thing in this country. Nor is it the apothecary only in England who would jump at it. The purity of the pures would not be scandalized at a little gentle repression of their numbers. We are all, physician, surgeon, and apothecary, too much in the situation of the Kilkenny cats.

But let us trace the rise and progress of the German pharmacopolist.

"About the age of fourteen or fifteen, the boy undergoes an examination before the pharmaceutic commission as to his acquaintance with languages, (Greek, Latin, French) the elementary mathematics, and general instruction, as history, geography. If he appears so advanced, that his special education can be commenced, he obtains a certificate to that purpose, and enters as lehrling or apprentice into a shop, for a term of three or four years. To almost every shop is attached a laboratory; and we must recollect, that with a German apothecary the student spends the years of his apprenticeship not merely in making up recipes, as is the custom here, but is engaged in the nicest investigations of modern chemistry, and works under the same circumstances that brought into action the neatness and accuracy of Klaploth and of Rose, that developed the transcendent powers of discovery possessed by Liebig and by Scheele.

The student having completed the term for which he had engaged himself to the apothecary his master, passes to the university, and commences attendance on the lectures of such professors as he considers best qualified to teach him what he wants. There is no curriculum made out; he knows the subjects on which he shall be examined; but he is left to acquire the knowledge requisite for passing when, where, or how he chooses, it being understood that he cannot leave his own country's university without special permission. For two or three years he attends the lectures on mathematics, physics, chemistry, botany, pharmacology, zoology, mineralogy, sometimes also anatomy and physiology; and generally works a year in the university laboratory, particularly if the university professor be

* Dublin Journal, July, 1837.

of eminence. When the student has thus spent at least five years in the acquisition of professional knowledge, he acquaints the pharmaceutic commission that he wishes to be examined, and is accordingly examined for two several days, and for more than two hours each day. The examination is rigidly confined to the physical and natural sciences, but is in these exceedingly strict. In chemistry, in botany, in the natural history of drugs, and the mode of preparing and compounding them, a degree of accurate knowledge is required, which it might prove very inconvenient to demand from many of our most admired teachers. If the candidate be approved of, he receives his license to hold a shop; his business then is, to try whether he can get one."

Apothecaries' Hall is nothing to this. How our students would relish such an examination as the German neophyte undergoes, we may, without much difficulty, guess. But this is not all.

"While the student has been thus reading for his degree in pharmacy, he generally attends the lectures of the professors to the philosophical faculty, and becomes, on the termination of his studies, Doctor in Philosophy. The majority of the leading apothecaries, whose acquaintance I was so fortunate as to make, are Doctors in Philosophy. In fact, the apothecary is as usually Doctor in Philosophy, as the physician Doctor in Medicine; the doctorate of the medicinal faculty being only a nominal degree, and quite distinct from the license to practise medicine. It is on this account, that persons who are rejected here, or in England, obtain doctorship in Germany so easily; but if they applied for a license to practise medicine, or to act as an apothecary, the result would give to them an idea of an examination completely new."

Some of *our* German doctors will, we are quite confident, take this kindly hint, and not content themselves in future with only the degree. Our M.D.'s of Erlangen and Göttingen, &c. will undergo the severe examination, merely for the purpose of establishing the national reputation in their persons. What a pity it is they did not know this long ago. They would not have been bantered on the "cheap and nasty" doctorate. But they may still avail themselves of the opportunity of obtaining distinction, and we feel assured that they will do so.

Let us enter the apothecary's shop.

"Our subject, who is now apothecary and Doctor in Philosopy, wishes to get a shop, and commence business. A shop can be obtained, however, only by one of two means, opening a new one, or purchasing one already established: both of these methods are restricted in a very remarkable manner. The government having compelled the student to a course of education requiring so considerable an outlay of time and money, is bound to provide that he can obtain a compensating return; and this is effected in a manner very well worthy of imitation.

To each district is allowed a number of apothecaries proportional to its population, averaging, in the greater part of Germany, one apothecary to 5,000 persons. The shops aie, of course, principally in the towns, and this might give rise to false impressions. Thus, in Giessen, with only 8,000 inhabitants, there are three apothecaries; but the surrounding country is very densely inhabited. Round Darmstadt the population is not thick, and therefore, though with 22,000 inhabitants, it has but five. Göttingen, with 10,000, has only two, the neighbourhood being but thinly covered with people. There are, however, real exceptions to this average. Thus, I was informed by Professor Dulk, that in Prussia Proper, and in Pomerania, owing to the scattered nature of the population, there is but one apothecary to 8, or 10,000. And in the Rhine-province of Prussia, the other extreme prevails, for there are not more than 2,000 people to one apothecary, owing to the law *having allowed,* during the occupation of the French, an unlimited number of shops, and many of them remaining still in existence.

In general, however, there are 5,000 people to one apothecary; and no person is allowed to deprive him of them; no retail druggists are permitted; none but an apothecary can sell medicinal drugs. The apothecaries themselves are not allowed to compete, at least by reduction of prices. Every year a price list for drugs is published by authority; and no apothecary is allowed to deviate from the prices contained in it, which are placed so, as to give a very high rate of profit, indeed much higher than could be obtained here.

When a district, from improvements of manufactures, or otherwise, increases in population, a corresponding number of new apothecaries' shops are opened by the government; or what is the same thing, permission is given to so many of those candidate apothecaries whose names are first on the list, to open shops in such places as require them. This is the one way of getting into business. The other is, that the shops in the already peopled districts, from time to time fall into the market, either from the death of their previous possessors, or from their possessors becoming legally incapable of continuing longer to trade in medicine.

In the case of a vacancy by death, the heirs of the late possessor have power to sell the concern, but the purchaser must be one of those who have obtained a license to open shop. And where the shop becomes void by the dismissal of the former occupant, he is always allowed by the government, though not entitled, to dispose of his interest in it to the best advantage, the purchaser being, as before, one of the qualified class.

The income arising from a shop being thus always respectable, and sometimes very considerable, the number of candidates, who are often taken from the most respectable bürgher families, together with the comparative rarity of a situation becoming vacant, raises the price of a good concern far beyond what, to our ideas, would appear its value. Thus in towns of from 6 to 8,000 inhabitants, it is usual to pay from 6 to £800 for an establishment. In larger towns, 12 or £1,400 is not uncommon; but in large cities, as Berlin, Dresden, Vienna, &c., the prices become enormously high. Indeed, I was assured by a gentleman of great eminence, and on whose veracity I can implicitly rely, that a short time ago, a shop, certainly one of the first in Berlin, was sold, and 60,000 thalers, equal to £9,000, paid down. I must add, however, that the establishments which bring such high prices are not mere apothecaries' shops; there is generally attached to them a factory of chemical preparations. The leading apothecaries are generally manufacturers of the nicer chemical substances, particularly the vegetable alcaloids; and the names of Merck, Winkler, Wittstock, and many others, are nearly as well known in the commercial as in the scientific world.

I mentioned that no apothecary is allowed to charge more for his drugs than the price regulated by the list; and as this price is quite sufficiently remunerating, he is forbid in the strongest manner from attempting to increase his profits by substituting an inferior article. There are in each state officers appointed, comprising the University Professors of Chemistry and Botany, who yearly submit to strict investigation the condition of every apothecary's shop in the district. We know that there is in this country a similar examination, but it is a mere matter of form; the results are never known, at least publicly; and the punishment is of too ridiculous a nature ever to be inflicted. In Germany, however, it is quite a different matter: each shop is separately the subject of a report, comprising the details of the size of the shop, states of drawers, glass cases, the number of rooms, the number of pupils, the nature of the library which the apothecary possesses, of the laboratory, the age, quantity, and condition of every single medicine."

This is rather sharp supervision, and even a German apothecary's shop may not always escape from it unscathed. Should matters not be quite comme il faut, the proceedings adopted by the authorities would appear to be somewhat summary.

"Submitted to so rigid an inspection, it need not create surprise that a shop should frequently give occasion to an unfavourable report of its condition. In that case the owner, if it be his first offence, is severely reprimanded. If he be a second time detected, a pecuniary fine is inflicted to a considerable amount, but varying in proportion to the importance of the shop, and the more or less grave nature of the offence. On his being a third time denounced, he is suspended, his license to hold a shop is removed, and the concern becomes legally confiscated to the state; but in fact, he always obtains permission either to make over the management, or altogether to sell his place to a person qualified to act, and of whom there are always many waiting such an opportunity: he is himself irrevocably dismissed from his vocation.

Having thus described the details of the regulations to which the apothecary in Germany is subjected, the result is capable of being conveyed in a very few words. He becomes the fellow-labourer, but not the rival, of the physician. His education is equal, though in a different path. His origin is as high; his income is as considerable; and he is received in general and in learned society on the same footing as any other man possessing equal property and information. If we look to any meeting of the German Association of scientific men, we find an independent section for pharmacy; and we likewise see that the great mass of the work of the chemical and botanical sections is accomplished by persons, who, if not apothecaries, were originally intended to be such, had not their talents and love of science carried them to a higher sphere of action."

There may be some advantages in such a system, but they are the advantages of despotism, and more than counter-balanced by those of freedom, with all *its* evils. If there is any principle which puts the human mind upon its mettle, and excites it to most vigorous efforts, it is that of unlimited competition. Continental despotism and centralization are incompatible with *its* existence, and the government affects to direct and to control every thing, from a dose of physic up to the making of a railroad. The consequences of systems so opposite as those of the Continent and of this country must be regarded not in detail, but in the gross—not in one solitary, but in a comprehensive aspect. When so regarded, the advantages will be found to preponderate greatly on the side of liberty, and when we look at England with her free institutions, and see the individual energy and enterprize, and the general power and resources she exhibits, we shall have no reason to be dissatisfied with her condition. It would be as impossible to introduce the German system of pharmacopolists here, as it would be to introduce the German system of bayonets; one indeed must come with the other, or not come at all.

We have not space, at present, to examine the comparative advantages of the two systems, viewed without reference to general considerations. This is of little consequence, as such a view would be merely speculative. But we may observe, that this has long been, and still is, a country of aristocratic institutions, habits, and feelings, and that our medical polity partakes of the national temper. As that undergoes, which it seems to be doing, a gradual alteration, our medical institutions must alter too. But the change, in partial as in general politics, will probably not be a very sudden one, for the party in favour of things as they are is strong both in numbers and in influence.

---

### Case of Hernia Pericardii. By T. Hart, A.B., Conservator of the Museum of the School of Medicine, Park-street.*

This, previously unrecorded, pathological condition occurred in an aged female subject, history unknown, taken into the Park-street school for dissection.

On opening the thorax, the pleuræ on both sides were universally united by old adhesions.

"The anterior mediastinum was occupied by a membranous pyriform sac

* Dublin Journal, July.

of considerable size, lying on and overlapping the pericardium, which was greatly distended, as well from the large quantity of fluid it contained, as from the hypertrophied condition of the heart, this organ being in a state of active aneurism. The sac contained fluid to the amount of three or four ounces, and was free and unattached in its whole extent, except at its upper and smaller extremity, where there existed a free communication between it and the pericardium, as proved by the following simple experiment:—By raising the sac off the pericardium, it was made to empty itself, the fluid passing into, and increasing the already distended condition of that sac; and again, on pressing on the pericardium from below upwards, the abnormal sac, in its turn, assumed its former dilated appearance.

The pericardium was next laid open by a longitudinal incision, and its communication with the abnormal sac brought into view: it was situated immediately at the point of reflection of the pericardium on the aorta, in one of those pouches, designated the sinuses of Haller, and presented a regular, well defined, circular orifice, freely admitting the introduction of a finger, around which the fibrous membrane of the pericardium formed a thick annulus of great strength, and then passed, considerably attenuated however, over the whole surface of the sac: the serous membrane passed into, and lined it throughout. The preparation of this very interesting case is now preserved in the Park-street Museum."

This does not appear to us to be strictly hernia of the pericardium, but rather a pouch communicating with the pericardium. There is no evidence to shew that any anatomical element of the pericardium had protruded so as to constitute the pouch, a necessary circumstance, we apprehend, for the existence of a hernia. But the case is curious.

---

On the Possibility of Transplanting the Cornea for Blindness from Disease of that Structure. By Dr. Bigger.*

The blindness from what is deemed incurable staphyloma is of course hopeless, unless some means of substituting a transparent for the opaque front of the eye can be devised.

Removal of that opaque part, and transplantation of a healthy cornea to its site have been thought of by several surgeons in Germany, and both essays and experiments have been resorted to with the view of recommending it. But on the whole, the evidence and authority both leaned against it, when Dr. Bigger, of Dublin, re-opened the inquiry, and prosecuted fresh and more extended experiments for its solution. The results have been published in our Irish contemporary, from whose pages we shall extract enough to put our readers in possession of Dr. Bigger's conclusions.

It is stated that Dr. Bigger has succeeded in rendering the removal of the cornea a safe operation, and easily practicable by a steady and dexterous hand. The following is his method of removing and of transplanting it.

Having fixed with a ligature the upper eyelid of the animal from which the cornea is to be taken, he introduces Beer's cataract knife (holding it horizontally, and at first directing it a little backwards, so as to insure its passing through all the layers of the cornea,) with its edge turned upwards, into that part of the cornea situated about a line or more from its most inferior junction with the sclerotic, and about the same distance external to the mesial line of the eye. He then pushes on the knife for the space of one or two lines, inclining the handle, so that the point of the knife may be brought forward, and caused to pierce the cornea again, at a distance as small as possible from the point of entrance. The knife should now be pushed on, when it will make as large a section as may be required,

---

* Dublin Journal, July.

which being turned down, is to be cut off with a pair of scissors. The eyelids are then to be closed, to prevent the escape of the crystalline lens and vitreous humour. The excised cornea should be placed on a slip of cork, and the curved needles, carrying very fine ligatures, (two, three, or four in number), should be passed through the cornea and the piece of cork. The latter, which has been chiefly used as a support to enable the operator to pass the needles through the tough layers of the cornea, should then be broken off, and the cut surfaces of the cornea should be keep moistened with some of the secretion from the eye. The surgeon then proceeds to perform the same operation on the eye to which the cornea is intended to be transplanted. Having done this, and closed the lids for a few moments, until the spasmodic action of the muscles of the eye diminishes, the operator proceeds to adapt the cornea to its new situation, and for this purpose, inserts the point of his needle carefully between the margin of the now prolapsed iris and the remains of the cornea, and pressing externally with the nail of the other forefinger against the point of the needle, so as to make it pass through the cornea without dragging or injuring the eye, draws out the needle. To accomplish the latter object, Dr. Bigger was often obliged to use a small forceps, and in this case, the thumb and finger nails of the other hand must be pressed closely and firmly against the cornea on either side of the needle, to obviate any injurious disturbance or dragging of the eye. The ligatures should then be carefully tied, and the ends cut off. Dr. Bigger has found two ligatures to answer the purpose quite as well as four. Finally, the operator clears away any lymph or blood which may have collected on the eye, and concludes the operation by smearing the eyelids with a little spermaceti ointment.

The unsteadiness of animals is a serious difficulty attendant on the operation. The great danger is—injury to the iris. Dr. Bigger performed many experiments previously to his return to Dublin, (he had been abroad). He then recommenced them, and instituted eighteen more. Of the earlier ones we need take no notice—the latter are deserving of more attention.

The subjects of the first and last we quote from our contemporary. Two rabbits were presented before an evening meeting of the King and Queen's College of Physicians, on the 18th of May last. They were examined with great interest by the members and visitors present, and the degree of vision which one of them evidently possessed, reflects the highest credit on the ingenuity, patience, and manual dexterity of the scientific operator. The results of these eighteen experiments were: in ten, the iris was injured; in eleven, the crystalline lens escaped; in seventeen, union took place between the implanted corneæ and the adjacent surfaces in forty-eight hours, so as to admit of the withdrawal of the ligatures, which are always a great source of irritation; in four, three ligatures were employed; in fourteen, only two, and with equally favourable results; in twelve, adhesion of the iris to some part of the cicatrix ensued; in one, sloughing of the cornea and destruction of the eye took place, an event which arose from the cornea being kept for half an hour without applying it, with the view of ascertaining how long it would be likely to retain a sufficient degree of vitality to enable it to unite. Dr. Bigger is inclined to think, that, generally speaking, a delay of this space of time would be prejudicial to the success of the operation, and that it may be always avoided by common dexterity on the part of the operator. Of the whole eighteen experimented on, sixteen recovered imperfect vision.

The difficulty of performing the experiment in such a way as to afford a chance of preserving the transparency of the implanted cornea, was a source of much disappointment to Dr. Bigger, and for a long period he could not succeed in devising any means for this purpose, until after his eighth experiment at home, when he discovered that much benefit might be derived from the local applicatiou of bichloride of mercury. A weak solution of this salt, gradually increased to the extent of three grains to the ounce of distilled water, and dropped into the eye three or four times a day, after the cornea had become adherent, was found by him to exercise an almost specific action in diminishing the opacity of the implanted cornea. He had made several trials with iodine and the nitrate of silver, but found that although they improved the appearance of the cicatrix, they did not appear to act upon the milky state of the cornea. The only caution necessary to be observed in using the corrosive sublimate is, to begin with a weak solution of it, and not to use it until the implanted cornea is perfectly united to its new connexions.

Of the two rabbits exhibited to the meeting, one, the first, had been left to Nature—the second had been treated with the bichloride of mercury. Nine months had elapsed since the operation in the instance of the first—ten weeks in that of the second. The advantages were immensely on the side of the latter, in which the power of vision seemed distinct, and nothing indicated the transplantation of the cornea but a slight line in the situation of the cicatrix, and some degree of conicality in the cornea itself.

There are a few desultory observations made in reference to the operation, which it is as well to notice.

*a.* One experiment accidentally convinced Dr. Bigger of the advantage derived from removing no more of the diseased cornea than is absolutely necessary, as the sound portion which remains may enact a very useful and important part in the reparative process. In this case, the iris was attached to the inferior angle of the cicatrix. Dr. Bigger has observed this in many of his experiments, and attributes it to the predominance of inflammation in the inferior part of the eye, a fact which he has noticed on numerous occasions."

*b.* "With reference to the applicability of the operation to the human species, Dr. Bigger observed, that he thought that in man the chances of success would be greater, at least so far as steadiness during the operation, avoidance of injury, and other obvious circumstances might contribute to that desirable end. With respect to the animal from which the cornea would be taken in the case of the human subject, Dr. Bigger has not yet decided, and invites the attention of comparative anatomists to this point of the investigation. The animal whose cornea he has found to make the nearest approach to that of man is the pig; it is, however, much thicker and coarser in its texture. In a spirit of just and humane feeling, he deprecates the removal of the cornea from the human eye, even when permitted for gain by the possessor; but thinks that a person afflicted with incurable amaurosis might be prevailed on to part with his pellucid cornea, which might be replaced by one taken from some of the inferior animals. He thinks, however, that the operation should not be sanctioned under any circumstances, when the patient enjoys even a tolerable degree of vision with the other eye, at least until our knowledge has been increased by further experiments and observations. He is of opinion that cases of blindness caused by small-pox, ulcers on the cornea, and ophthalmia not affecting the deeper structures of the eye, would be the most favourable for operation."

Such are the main portions of this, not uninteresting paper. Our ophthalmic brethren will judge for themselves. We must say that we do not anticipate much from the operation in the human subject. It is one thing to remove the cornea from the sound eye of an animal, and another to remove it from a staphylomatous one. This will augment the difficulties of the operation in the human subject.

Its dangers are not inconsiderable. They would appear to be—adhesions of the iris to the cicatrix—injury of the iris—escape of the crystalline lens—opacity of the transplanted cornea—deep-seated inflammation of the eye—sloughing of the transplanted cornea.

When we sum up the many difficulties and the considerable dangers, we fear we must not reasonably anticipate from the operation any more than the *possibility* of some benefit in a rare and lucky case. But it will give us sincere pleasure if our own unfavourable anticipations shall turn out to be unfounded. Whether the operation succeeds or not there can be but an unanimous opinion with respect to Dr. Bigger. He deserves the highest praise for his philanthropical zeal.

---

On Violent Pulsations in the Epigastric Region. By W. Faussett, A.B.*

This is rather a long, we might say rather *too* long a paper. Professing to discard speculation, the author very liberally speculates. We may safely venture to shew in a few extracts all that is of consequence.

It is well known to our readers that Dr. Baillie first drew attention to the not unfrequent occurrence of epigastric pulsation independently of any aneurysmal dilatation of the aorta. Since Dr. Baillie's time most physicians and surgeons must have had opportunities of verifying the general correctness of his observations. But Mr. Faussett quarrels with them, and substitutes, as more correct, the following description of his own.

"The complaint is most apt to occur at the middle period of life. Dr. Baillie knew of but one or two instances of it in persons so young as thirty. I have, however, known three cases of very severe epigastric pulsations occurring, one of them at twenty, the others between twenty-three and twenty-five, all of them in unmarried females, and in the better order of society. According to Dr. Baillie's experience, it was more commonly to be found in men than women; but this, I believe, is the reverse of what other practitioners have met with. The rich and poor are alike subject to it. The digestive organs are always deranged in their functions; the bowels are torpid, and the feet cold: in some cases, there is considerable constitutional derangement, with headach, vomiting, emaciation, lowness of spirits, and prostration of strength. There is, *in every case*, pain, on pressure of the pit of the stomach, or towards the umbilicus; and, *in every case*, a considerable fulness, sometimes amounting to tumour. The pulse at the wrist is usually perfectly natural and regular in its beats; in a few cases, however, it is accelerated. The pulsations are marked by different degrees of strength in different individuals, and vary also in the same individual. They come on, most violently, in the afternoon, or some time after dinner, there being, in some degree, a remission in the morning. They are exceedingly distinct in the erect posture, but perhaps still more so in the horizontal. They are frequently visible to the eye, often indicating, as Dr. Baillie observes, the boundary of the artery as far as the umbilicus. In some of the severer cases, the degree of vital depression is truly remarkable; and where the head is engaged, there is not only uneasiness complained of there, but a sense of weight, fulness, tightness, or throbbing. In one case, where the epigastric pulsations were thought lightly of, or indifferently treated, the patient, who was personally known to me, died of hydrocephalus; and it is worth remarking, that Dr. Baillie's ill-fated patient, already mentioned, and in whom, perhaps, the pulsations occurred in a more moderate degree, ultimately became the subject of paralysis."

We have seen several cases, as well as our neighbours, of epigastric pulsation. Certainly pain at the epigastrium has *not*, in our experience, been universal. From Mr. Faussett's observations, and from the cases he narrates we suspect that the subjects of the affection which he describes have been principally females affected with those anomalous hysterical symptoms, which ape all maladies. In such females, epigastric pulsation, as well as cardiac palpitations, are frequent, and in them, too, of course there is epigastric tenderness. We say, of course, because such patients have tenderness in any part to which attention is directed. We offer these observations

---

* Dublin Journal, July 1837.

that our readers may make what allowances are necessary in reading Mr. Faussett's descriptions.

He enters into reasonings more elaborate than conclusive, and more hypothetical than satisfactory, to prove that in cases of epigastric pulsation there is congestion or chronic inflammation of the viscera, implicating the ganglionic nerves and plexuses—and that such congestion plays a main part in the production of the morbid pulsation.

This view is not entertained for nothing. It is the foundation of his practice. The following is an account of *it*.

"If we admit this view of the subject, the treatment comes easily to be inferred; but the fact is, that this view of the complaint, imperfect as it is, has in itself been derived in a large measure from the results of treatment. '*Ubi irritatio, ibi fluxus est,*' was an axiom of the illustrious Celsus: and in so many words affords a rationale of cure, wherever irritation has been followed by determination of blood and congestion, or inflammation. The plan of treatment which I wish to refer to is as follows: local bleeding by cupping and leeching, (a combination of these, leeching first and *then* cupping, answers the purpose well.) In a few cases general blood-letting is necessary, viz., those in which the symptoms are severe, and local abstraction of blood has been employed in vain, or where the pulse seems to warrant the practice; or without this criterion, where there is a sense of tightness, fulness, or uneasiness in the head,—in any modification of such cases, venæsection from the arm to a moderate extent, may be practised with safety, the relief afforded the patient will be striking, and the blood will often be found buffed, and even cupped. After local bleeding, counter-irritation by means of antimonial ointment, or the croton oil, rubbed over the epigastrium, or between the shoulders, may be employed with benefit. The state of the digestive organs demands particular attention, if an obstruction be suspected in the arch of the colon from an accumulation of fæces there, or in any other part of the intestines, a tolerably active purgative had best be first exhibited and mild aperients afterwards. But as soon as local bleeding has been *first* practised, and the bowels moved, we should at once have recourse to the use of blue pill, combined with sedatives and James's powder, or hippo, and give it twice or thrice daily, until the mouth becomes slightly affected. The inspissated juice of cicuta, or henbane and hippo, are what I have conjoined with blue pill, and derived the greatest advantage from. If the urgency of the symptoms demand it, and it be thought desirable to expedite the effect of the mineral upon the mouth, mercurial inunction either to the inner part of the thighs, or over the abdomen, should be resorted to, for it will invariably be found, the moment the gums become tender, and seldom before this period, that all the symptoms are mitigated. A strict regimen must be also enjoined; meat, wine, and stimulants, must be forbidden, and contrary to what is imagined, and usually practised, the patient's diet should be made to consist of milk, and farinaceous vegetables; barley, arrow-root, sago, &c. &c. In the course of a few days, (averaging at about a fortnight,) and often contemporaneously with the mouth becoming sore, it will be found that the epigastric pulsations have ceased, that the appetite and digestive functions have improved, the patient's spirits returned, and a general renovation taken place in his state of health."

This treatment, it must be confessed is somewhat active—local, nay general bleeding — counter-irritation — salivation—low living are the means which hang on his inflammatory theory. And, considering that the cases are, according to his own shewing, principally those of young unmarried females, such means are not to be lightly used.

Fór our own parts, we must own that in every case of epigastric pulsation we endeavour to discover the cause in *that* case, and, having discovered, to remove it. Such treatment must necessarily be discriminative, and we should pause before we applied the powerful remedies advised by Mr.

Faussett to females of a "hysterical" habit. But we leave the matter to our readers.

---

### MEDICINE IN AMERICA.*

The American Periodicals before us are the two Numbers of the American Journal of the Medical Sciences—one for February and one for May of the present year. These are all we have received for some time, and our notices of American Periodical Literature must be limited to their contents.

#### I. CASES OF ANEURYSM, CURED BY OPERATION. By M. MORRISSON, M.D. &c. &c. of Buenos Ayres.†

Dr. Morrisson relates six cases of aneurysm, cured by operation. He relates none of failure; either, we suppose, because none, in his hands, have failed, or because they were not *so* interesting as the successful ones. Of the six cases which he does relate, one was an instance of femoral aneurysm at the upper part of the middle third of the thigh—one was a femoral aneurysm just where the femoral artery passes through the sheath of the triceps—two were cases of popliteal aneurysm—one was a case of inguinal aneurysm—and one was a case of aneurysm of the root of the right carotid and arteria innominata.

The first and the second cases are the only ones that require particular notice.

1. *Femoral Aneurysm—Femoral Artery tied just below the origin of the Profunda.*

*August* 10, 1832. Don Luis Sagari, æt. 24. The tumor was large and diffusive, and situated on that part of the artery, which is in general the point of election made by surgeons for applying the ligature for the cure of popliteal aneurysm. The tumor pulsated and became very painful when prest upon. There was a difference of opinion among the surgeons who had already seen the patient, as to the propriety of tying the femoral artery above or below the *arteria profunda.* This man had been confined to bed, and been subjected to the plan of Valsalva, and local pressure had been made on the tumor with a bandage and compress, without any permanent benefit. The patient was reduced in flesh, in spirits, and of a consumptive family. The inguinal glands of the affected side were very large, a circumstance which determined Dr. Morrisson to operate below the origin of the profunda. This he did on the 20th of September, and the patient did well. The ligature came away on the 22nd day.

The interest of the case consists in the non-occurrence of secondary hæmorrhage, after the operation of tying the femoral artery, a short distance below the origin of the profunda. But the spot at which this vessel springs from the trunk is so various, that too much stress must not be laid on such a case.

2. *Successful Operation beyond the Aneurysm.*

*Aneurysm of the Root of the Right Ca-*

---

* We are extremely anxious to put our English readers in possession of the valuable facts which medicine owes to our brethren in America. But our supply of the American Journals is both scanty and irregular, and it is impossible to do justice to them or to their noble country, unless some better means of transmitting their periodicals and works are hit upon. Ever and anon, it is true, there comes a pamphlet or a book, by post, with the moderate charge of two or three or four pounds sterling. A little consideration would convince our American friends that this is killing us with kindness, and that no Editors could survive such doses *if* they swallowed them. The fact, indeed, is, that we cannot and we do not swallow them, and, reluctantly enough, we are obliged to return such packets to the postman.

We intreat American editors and authors to look to this. It is for the advantage of science that their labours should be known in the Old World.

† American Journal for Feb. 1837.

*rotid and Innominata. Right Common Carotid successfully tied.*

On the 4th of November, 1832, Dr. Morrisson was summoned to Don José Hurruz, æt. 42, who had more or less been confined to bed for nine months. He had pain in the cardiac region; difficulty of breathing, which was increased to a painful degree by walking a distance of a few squares; rheumatism of the right shoulder, and of the muscles of the neck of the same side; hemicrania of the right side; and a large pulsating tumor extending itself upwards from within the cavity of the thorax, and obliquely outwards behind the sternal extremity of the *sterno-cleido mastoid* muscle.

The tumor, when pressure was made on the carotid above it, did not diminish perceptibly, but the pulse at the right wrist became fuller and stronger. When pressure was made on the right subclavian, the tumor pulsated more strongly, as likewise did the temporal and facial arteries of the right side. From all these circumstances, Dr. Morrisson concluded that the case was one of aneurysm of the root of the right carotid and arteria innominata.

The same opinion was subsequently formed again in consultation, between Dr. J. M'Donnell, and Don Mariano Bico, the Surgeon-General. The nature of the case was represented to the patient, and the advantages and disadvantages of the operation of tying the common carotid artery *beyond* the tumor were fairly represented to him. He determined to take the chance which the operation gave him, and this was performed on the 8th instant, in presence of the gentlemen just mentioned. He was first bled, and had a dose of salts.

The operation was commenced by making an incision, extending from a point on a line with the top of the larynx downwards along the internal margin of the *sterno-cleido mastoid* muscle, as far as possible without endangering the superior margin of the tumor. The dissection being continued, the *homo-hyoideus* was exposed, and the sheath which involves the primitive carotid and jugular vein, laid bare. The patient now became pulseless, and to revive the powers of life a draught of undiluted wine was administered. Upon the recovery of the patient from this state, the sheath of the great vessels was raised with a dissecting forceps, and divided with a scalpel. An aneurysmal needle, armed with a flat silk ligature, was passed under the artery, and the vessel secured by the ligature. The wound was brought together by the interrupted suture, dressed with adhesive straps, carefully bandaged, and the patient put to bed.

In the afternoon the patient complained of being much distressed by hollow sounds in the right side of his head; the pulsation in the tumor was tremendous, and by reason of communicating its motion to the shirt collar, the number of pulsations could be taken at some distance from the bed: the artery at the wrist being full and hard, and his skin hot and dry, Dr. M. bled him to 16 ounces.

Nex day, the tongue was loaded, the pulse strong—*salines.* In the evening, it became stronger, and was 110. *V.S. ad* ℥x.

Saturday 10th, passed a restless night, suffers from pain in the right side of the head; pulse 100 and soft. The saline cathartic to be repeated.

11th. The tumor pulsates strongly; some tenderness in the region of the wound; pulse 100; skin moist.

12th. Dressed the wound, the greater part of which had healed by the first intention; pulse 98 and soft.

13th. Pulse 94; wound doing well.

14th. Pulse has risen to 108. V.S. ℥viij., which produced fainting.

15th. Pulse 100 and strong; twelve leeches applied to the region of the heart.

16th. Digitalis and nitre were prescribed, but in consequence of the constitutional disturbance produced by their exhibition, they were discontinued.

17th. The tumor was tender to the touch; from this date the pulsation in the tumor became weaker, the tumor itself began to harden and to diminish in size; the only symptom that afterwards required treatment was the rheumatic pain of the back of the neck and shoulder.

About the beginning of December the patient sat up to his meals; and on the ninth of the same month, being the thirty-second day from the operation, the ligature came away.

Hurruz, after his convalescence, laboured hard at the making of charcoal. Dr. Morrison saw him from time to time, and he continued well until the latter end of June, 1834, when he complained of an oppression in the chest, accompanied with slight cough. On the 4th of July following, after walking some distance, he fell down dead.

*Dissection.* This was made next day in the presence of Dr. M'Donnell.

"The sternum, together with the cartilages of the ribs being raised, the right subclavian artery was traced to its source, at which point it was partially dilated. The *arteria innominata* was double its ordinary size, and studded with spicula of ossific matter.

The right carotid, from its origin to the point to which the ligature had been applied, was dilated into a sack, which was plugged up with a dense fibrous deposit. The portion of the tumour which was within the chest, was much more voluminous than that which rose into the neck.

The anterior surface of the thoracic portion of the tumour was firmly adherent to the sternum. There never was a more splendid specimen than this, showing the manner in which Nature cures this formidable disease when assisted by art.

The lungs were free of adhesions, and of a healthy appearance; the heart itself presented nothing uncommon in its external aspect, but upon dissection it exhibited evident marks of inflammatory action, particularly in its ventricles; the semi-lunar valves at the origin of the *aorta,* were indurated from a deposit of ossific matter; the arch of the aorta was completely ossified and considerably dilated. The pericardium contained from 8 to 10 ounces of serum."

It appears that the preceding was an instance of aneurysmal dilatation of the arch of the aorta, aneurysmal dilatation of the innominata, and an aneurysmal sac at the origin of the right common carotid; the coats of the vessels were diseased, and the semi-lunar aortic valves were more or less ossified. We must be excused from yielding entire assent to the declaration that the lining membrane of the heart was inflamed. No unequivocal effects of endocarditis are mentioned, and it is possible, nay, from the circumstances, it is probable that the "inflammatory appearances" consisted merely in redness, the result of staining.

The case must, on the whole, be regarded as favourable to the operation of tying an artery beyond an aneurysmal tumor. The aneurysm at the root of the carotid would certainly appear to have been cured, or to have advanced far towards cure, and we must presume from the details of the case that this was greatly owing to the operation.

At the time when Mr. Wardrop directed public attention to the ligature of arteries beyond an aneurysm, we strongly supported the proposition that, of all arteries, the common carotid was most favourably circumstanced for the performance of the operation. We argued, too, that when the disease consisted of an aneurysmal sac, it was more likely to be cured, than when it was simply an aneurysmal dilatation, coagulation of the blood taking place with more facility in the former case than in the latter.

When the propriety of extending the operation to cases of dilatation of the innominata was insisted on, we took occasion to express our suspicion that it would be found inapplicable to that disease. The flow of blood through the innominata and subclavian would continue, and there would not be sufficient retardation of the circulation to permit the contraction of the dilated vessel.

The case before us bears out these views to the letter. The operation of tying the common carotid *cured* the aneurysmal sac between the ligature and heart—but did *not* cure the dilatation of the innominata.

The case too proves another thing—the necessity of ascertaining, so far as auscultation will enable us to ascertain it, the condition of the aorta, the heart, and its valves, prior to performing operations for aneurysm especially for aneurysm in the cervical region. It is quite evident that the existence of more and of other disease than is apparent, or than we count on, must materially diminish the chances of success from any operation on the great vessels—and not only must it diminish the probability of ultimate success, but it must increase the immediate risk. Yet we have seen surgeons determine upon tying the larger arteries without ever ascertaining or making any precise inquiry into the condition of the heart and the aorta; an omission pregnant with danger.

### II. Observations on the Treatment of Puerperal Convulsions. By W. Denny, M.D., of Ellicott's Mills, Baltimore County, Maryland.*

Dr. Denny displays a degree of candour, which, unfortunately for medicine, we do not often see—he not only relates unsuccessful cases, but he forcibly points out what he deems his fatal errors in their treatment.

His object is to shew that depletion by the lancet may be carried much too far in cases of puerperal convulsions. His paper is a long one, but two or three extracts will give the *points*, and those are what our readers want.

He first cites the opinions and the rule of action of Professor Dewees:—"If it should be asked," says the Professor, "what quantity of blood should be drawn in any given case? I answer: I do not know by ounces—I bleed until I abate the severity of the fits, or until I arrest their repetition."

With such emphatic words ringing in his ears, Dr. Denny met with cases of puerperal convulsions. Six occurred to him. He details them all; but the following summary which he himself gives of them, and the one which we shall quote to illustrate his commentary and to explain it, will be sufficiently instructive.

"In subjecting to review my own proportional amount of puerperal cases, complicated with convulsions, I have sins of commission and omission to confess. But an impartial witness will present to the tribunal at which he appears, the whole truth he possesses in the matters before it; and if the facts detailed bear the inferences I draw, and shall lead but one practitioner in a single case to profit by my exposition, I shall not have intruded on these pages in vain.

In my first case, I endeavoured to bleed *enough*, but was prevented by the circumstances stated, from succeeding. No meddlesome midwifery, however, was practised. This patient may be considered to have died from the repletion or excitement which caused the convulsions, not having been reduced by sufficient detraction of blood.

The second case presented indication that the apoplectic species was at hand. This was correctly and timely met by a free venesection. My first error was to leave this patient during the temporary calm, in place of waiting to strangle reaction at its inception. Reaction came on uncontrolled; but, because of the first bleeding, the form of attack was reduced to epileptic. The use of the lancet, a second time, gave earnest, on my part, of the employment of energetic measures. Another convulsion was the signal to bleed again freely—too freely! This was the second error, but it was in obedience to the rule to bleed according to the severity or repetition of the fits. Believing that depletion had been carried to its utmost justifiable extent, I followed the ideas of those who look on speedy delivery among the means of combating the disease. I reasoned thus:—Gestation is one of the causes of puerperal convulsions—parturition is its only favourable termination. Now, as there are here no natural pains of labour, I must deliver artificially, which was done without hurry or violence. This interference constituted error the third.

There was no ground to anticipate danger in the third case, as I recognized no premonition. The lancet was used freely in the shortest possible period, after the access; I bled till the

* American Journal, Feb. 1837.

approach of syncope. What began as impending syncope, ended in convulsion. An improvement ensued, viz. spontaneous delivery. The third convulsion was severe, but it came on with signs of subdued arterial action, and I bled no more. In the propriety of this forbearance two medical friends, who visited the patient with me afterwards, fully concurred.

Sustained and commended by their authority, it would not be strange if, by this case, I should have gained some reputation among the friends of the lady; it would not be as much so, if I laid claim to the sustentation and commendation bestowed. But candour obliges me to see one error, viz., I bled a little too far or too rapidly, and, when I threw aside the rule of treatment quoted from Dr. Dewees, and withheld the lancet, it was under the stern and intelligible facts before me, that syncope had ushered in the second convulsion, and that syncope was the result of depletion; consequently, to deplete further, would be to aggravate the condition of the circulation, on which syncope depends, and I might fasten the convulsions upon her, instead of removing them.

In the fourth case I suffered the precursor to pass without adopting measures of prevention, practised ultra-depletion, and then used the forceps. Here are three errors.

My treatment of this affection was thus far unsuccessful and unsatisfactory. I had lost my confidence in the infallibility of the lancet, and was not in possession of any substitute. I had no right to doubt that bleeding was an important remedy; but why has it failed in my hands, under a rule of treatment which has obtained for it signal triumphs in the hands of others? Is there not danger from excess in bleeding? We have ceased to prescribe other remedies for the names of diseases, and we regulate their application to conditions of the system. Why shall this agent be excepted from the improvements of our day? Shall we shed blood in puerperal convulsions because of their severity or recurrence, when it is known that a similar train of symptoms is often the consequence of hurtful waste of the vital fluid?

When the fifth case presented itself, under circumstances which allowed me to act free from agitation, I depleted with more legitimate aim, viz., to reduce the vigour of the circulation into moderate bounds, and to avoid carrying the practice to an opposite extreme. Dr. Copland says that the arteries of the lower extremities pulsate with diminished force and volume, while those of the upper and head are strong and active. But I have found the radial pulse convey no information of the excitement present. In the fifth patient this was the fact. I therefore turned to the signs afforded by the central organ of the circulating system, and found a guide. The treatment has been detailed, and the favourable issue of the case seems to confirm my reasoning.

In the sixth case, the same therapeutics procured the same happy result."

It is the fifth case which we have singled out for narration, because it was successful, and because Dr. Denny conceives that it exemplifies more appropriate treatment than unflinching abstraction of blood will prove.

*Case.* On April 4, 1835, Dr. D. was summoned to a German woman, Mrs. — æt. 20. It was her first pregnancy; she was bloated, and lay comatose. As the husband and relatives spoke little but German, Dr. D. was unable to obtain accurate information.

"I gathered from them, however, that she had had eleven convulsions. I determined to wait for a repetition, to decide whether the attack might not turn out to be hysterical; if so, I thought, there is but little danger, and if epileptical, she can scarcely be saved by any means. The pulse at the wrist was small, and almost countless; but the heart impinged powerfully against the parietes of the thorax. She soon had another fit, which I recognised as the more formidable species. Under all the circumstances, my judgment was cool and deliberate. I drew blood to command the action of the heart, but guarded cautiously against syncope, or that collapse of the powers of the circulation which may have resulted from ultra-depletion in the 2nd and 4th cases above detailed. In depleting, therefore, I took the action of the heart, not the severity or repetition of convulsion as my index. During the flow of blood she had a thirteenth convulsion, and I noticed that increased tumult of the heart preceded its approach. Having closed the vein, I observed the thumb of one hand contract quickly but feebly towards the palm, and, coincidently, the heart pulsated again more violently. I drew two or three ounces more of blood, stopping as soon as the inordinate palpitation subsided. A bye-stander remarked, that she was sure the last bleeding prevented another fit, as she observed a flickering of the features, such as had preceded the previous ones; I had looked at the thumb, she at the countenance, both had discovered signs of an impending paroxsym, but each was ignorant of the indication observed by the other.

Labour-pains now came on vigorously, and in about four hours she gave birth to a still-born infant; the secundines followed, and the uterus contracted duly. I ought to mention that the head became stationary for some time near the outlet, that I applied the forceps, started it from its resting place, and then allowed nature to complete the process. During these four hours she moaned and uttered complaints in her native tongue, so that I could not judge by her ejaculations, whether her intellect was impaired or not. She did not reply, however, to some questions put to her by her husband, at my request, with the view to discover the return of mental energies.

Immediately after delivery, she fell into a deep and quiet sleep, her pulse somewhat feeble; stroke of the heart distinct and normal; respiration full and equable. The next morning I found her better than I expected, and nothing occurred during the two or three weeks of getting up to embarrass perfect recovery."

She has since been happily confined, and given birth to a living female child. The following short passages embody our intelligent and candid author's conclusions, with respect to the line of practice to be followed.

" I accord, then, with the magnates of obstetrical science, in the great importance of bleeding in the attack of puerperal convulsions of entonic origin. I would practice it, however, with a strict watch over the radial and carotid pulsations, as well as the stroke of the heart. My patient should keep a horizontal posture. I would draw blood at first freely, but slowly, and with intervals of interruption towards the last. The lancet is the grand remedy; there may be adjuvants; the cold dash to the head might be placed next to the lancet. Some have recommended secale cornutum. It might be given here where the parturient efforts were inefficient, and other circumstances did not forbid its use."

" In cases where the convulsions occurring after the commencement of labour have been subdued, and labour-pains do not recur, I should certainly prefer the ergot, circumstances permiting its use, to pure artificial delivery, for of this I have seen enough to deter me from its repetition.

The fœtus being delivered, I would take great pains to ensure due contraction of the uterus, as preventive of hurtful sanguineous loss. I would enforce on the patient and her nurse that quietness from every exertion was of the highest importance; and finally, other circumstances being equal, she should be more guarded in her diet, and postpone her rising from bed to a later period than in ordinary uncomplicated parturition."

### III. Supposed New Views of Certain Dislocations. By S. Annan, M.D. of Baltimore.*

1. *Reduction of a Dislocation of the Head of the Humerus into the Axilla, by the Foot on the Acromion.*

After examining the muscles which oppose reduction of a dislocation of the head of humerus into the axilla, and criticising the usual methods of applying

* American Journal, February, 1837.

extension and counter-extension, Dr. Annan proposes his own plan in the following terms.

"The best plan of reducing this dislocation we believe to be to place the patient on his back on the floor, or on a bed or table. The floor is preferable, as the surgeon can apply the forces more conveniently. The surgeon should take a seat beyond the head of the patient, place his foot on the top of the shoulder, and, grasping the wrist, draw the arm towards him, which, if the patient was in the erect position, would be directly upwards. The foot pressing upon the spine of the scapula and the clavicle fixes the socket immovably, and thus we have the counter-extending force applied more advantageously than it can be by any other method, while the extending force draws the head of the bone from its situation below the glenoid cavity in the direction best calculated to restore it to the place from which it has escaped; and at the same time the point of the acromion process acts as a fulcrum to throw it out of the axilla, and afford it a favourable opportunity to slip over the projecting inferior margin of the glenoid cavity. The muscles too, which usually offer the most resistance, especially the deltoid and supra spinatus, are relaxed in the greatest possible degree; and indeed to that extent that they make no resistance whatever. The supra spinatus, if it acts at all, will assist in drawing the displaced bone in the proper direction, for restoration to its natural position. Another advantage is, that counter-extension and extension are not both applied to any of the muscles. The force which fixes the scapula, being made to press upon the top of the shoulder, does not act upon any of the muscles concerned in the dislocation, and the only muscles upon which the extending force acts at all, and which will make some resistance, are the latissimus dorsi, and teres major, and the inferior margin of the pectoralis major, where it arises from the cartilages of the fifth, sixth, and seventh ribs, and the lower part of the sternum. The resistance of these is insignificant."

Our able contemporary, the editor of the American Journal, shews very decisively that this mode of reduction is not new. It was proposed by M. Mothe, in his Mélanges de Medecine et de Chirurgie, published in 1812. By one of his methods (for he recommends two), the patient is laid on a low bed, near its edge, with the dislocated arm on the outer side. The surgeon seated on a low chair above the patient's head, seizes the dislocated arm, draws it upwards in a line parallel to the axis of the body, whilst at the same time he fixes the scapula with his foot placed upon the acromion process.

This plan, as our contemporary observes, is identical with that of Dr. Annan.

We have never seen this mode of reduction put in practice, and we cannot therefore speak to its advantages or disadvantages in comparison with those of the ordinary procedures. But we are sure, that, with proper care, the latter are in common cases very simple, very successful, and by no means hazardous—and, in old unreduced dislocations, it remains to be seen whether the plan of MM. Mothe and Annan is possessed of any real superiority. It gives us, however, another string to our bow.

2. *New Mode of reducing a Dislocation into the Ischiatic Notches.*

If there are any of our readers who have forgotten their anatomy, it may be worth while hinting that the two sacro-sciatic ligaments, with the spinous process of the ischium, divide the great sacro-sciatic notch into two foramina—the upper larger—and the lower smaller one. If the head of the femur is dislocated into the former, the limb is a little, and but a little shortened—if into the latter, the limb is considerably lengthened. The first of these two kinds of dislocation is more frequent than the second.

If there are any of our readers who have not by heart Sir A. Cooper's Treatise on Dislocations, it may be worth while hinting that that veteran surgeon recommends for the reduction of the first kind of dislocation, we may say (for he does not, remedially speaking, distinguish them) for both kinds, the plan we are about to mention. The patient to be placed on a table on his side—the pelvis to be fixed by a girth between the pudendum and inner part of the thigh—a napkin to be carried under the upper part of the thigh, and either pulled up by an assistant, or raised by being passed across his shoulders, while he forcibly elevates his body;—extension from above the knee, the dislocated limb, while extended, being directed across the middle of the sound one.

To this plan Dr. Annan urges several objections, which we need not enter on, but content ourselves with quoting the method which he advises in its stead. We should observe that this method is advanced by its author as peculiarly applicable to the dislocation into the lower notch, that in which the limb is lengthened.

"The principle of our method is the same with that recommended, by Sir A. Cooper, for dislocation into the foramen ovale, viz. lateral extension to the exclusion of longitudinal. The patient should be laid upon his face on a table; the pelvis secured by a band passing round it, and going off laterally from the sound side to be fastened to a post or a ring fixed in the wall; another band should be put round the upper part of the thigh of the injured limb, which should be given to the assistants, who are to make the extension; or have the compound pulley fastened to it, if it is to be employed. If the limb is lengthened, the bands should be so adjusted that the extension can be made a little upwards as well as laterally, which will move the head of the bone in a direct line towards the acetabulum, and by a gentle rotation outwards it will be replaced. Sometimes it may also be necessary to push the limb upwards.

"When the limb is shortened, which happens where the luxation is into the upper notch, the apparatus should be adjusted in the same general manner; but the bands, instead of being directed downwards and upwards, should pass off from the body at right angles with the trunk, and the extending band should only be tightened, so as to act as a fulcrum upon the upper part of the thigh. The ankle of the dislocated limb should then be taken hold of, and be drawn over the sound limb, which will force the head of the bone out of the notch, and make it describe the segment of a circle, and pass a little downwards in the direction of the acetabulum, and thus be speedily replaced. Care must be taken that the extending band is sufficiently tightened, and also that it does not yield; otherwise the drawing of the leg across the other will only move the head of the bone in the notch, as if it was a joint. If lateral extension only was employed in this case, the head of the femur would be drawn out of the notch; but it would be upon the dorsum of the ilium, above the acetabulum. Whereas, by simply drawing the limb laterally as much as is required to make the extending band serve as a fulcrum, and then using the leg as a lever, the head of the bone is not only forced laterally, but moves downwards, and must necessarily pass into its socket.

"Besides fulfilling the two general indications of fixing the socket, and causing the head of the displaced bone to move towards its natural cavity, (there are no muscles that require to be particularly relaxed,) if the pelvis is examined, it will be found that instead of its being necessary to raise the head of the femur *up over* the edges of the acetabulum, there is a continued descent along an even surface, thus affording the greatest possible facility for reduction. It is very apparent, too, that when longitudinal extension is made, it must be much more difficult to move the bone laterally than when it is dispensed with."

It is proper to observe that this mode of operating has not hitherto been put to the test of experience. It is right that surgeons should be made acquainted with it.

IV. SUCCESSFUL LIGATURE OF THE INTERNAL ILIAC ARTERY, FOR ANEURYSM EITHER OF THE GLUTEAL OR THE ISCHIATIC. By VALENTINE MOTT, M.D. &c.*

The reputation of Dr. Mott, as an operator, stands high in the Old World and the New, and we regret to hear that ill health has compelled so able a surgeon to retire for awhile from the exercise of his profession. May that retirement be brief.

Dr. Mott has added another laurel to the crown already on his brow. He has tied the internal iliac artery with success. That operation has been twice performed for gluteal or ischiatic aneurysm, with the happiest effects—once by Dr. Stevens—and once by Dr. S. P. White, of Hudson, New York. This, therefore, makes the third fortunate case.

*Case.* New York, December, 1834. Richard Charlton, the patient, is a coloured man, born in this city, and about 38 years of age. He has worked in a grocery store. He first felt the symptoms of his disease in the Summer season of 1832;—during the cholera then prevalent he had a diarrhœa, and while making frequent straining at stool, perceived a swelling and pulsation in the right buttock, which has gradually increased until this time. It is now about the size of a goose egg, and contained only fluid blood.

On the 29th December, 1834, at noon, Dr. Mott proceeded to tie the right internal iliac artery, in the presence of Drs. I. Kearney Rodgers and A. E. Hosack, and assisted by Drs. Vaché and Wilkes. The incision, which was fully five inches long, extended from a spot on a line with the umbilicus, about midway between the linea alba and the anterior superior spinous process of the ilium, to within half an inch of Poupart's ligament, and then curved forward an inch over the course of the spermatic chord. The operation lasted about 45 minutes, owing to the almost irrestrainable intractability and frantic restlessness of the patient. His great straining and jactitation caused the operator to make a small opening in the peritoneum, whilst separating it from the iliacus internus muscle. The peritoneum and intestines being drawn up and supported by a large curved spatula, the internal iliac artery was readily seen, crossed by the ureter, which was easily pushed aside. The filamentous tissue was quickly separated by the fingers from about the vessel, and the ligature conveyed under it by the American needle. At the moment of tightening the knot the hand was applied to the tumour, in which all pulsation immediately ceased, and which itself almost entirely disappeared directly after. The patient, being put to bed, took twenty drops of a solution of morphine, and in the evening was easy.

*December* 30. Had a good night's rest, and was comfortable in the morning. Some excitement coming on early in the afternoon, he was bled from the arm to about ℥xviij., and took a solution of sulph. magnes. in divided doses. *Evening.*—Much easier; salts had not operated. Directed an enema, and applied a strip of blister plaster around the wound.

He passed a good night, and next day he was doing well. The enema gave rise to too frequent stools, and an opiate injection was required on the 1st of January. On the 3d, although the abdominal tenderness was gone, the blister was reapplied, and panada and arrow-root were allowed.

On the 9th, the sutures were removed from the wound, which was much closed.

On the 42d day the ligature came away.

The patient recovered completely, and having lived as a domestic in the family of Dr. Mott for seventeen months, he then quitted it to become a coachman to a gentleman of New York, which situation he held in perfect health at the date of the report, in Nov. 1836.

V. CASE OF CONGENITAL ENLARGEMENT OF THE TONGUE REMOVED

* American Journal, May, 1837.

by Operation. By Thomas Harris, M.D., Surgeon to the Pennsylvania Hospital, &c.*

*Case.* R. R., aged 19, consulted Dr. Harris, in May 1835, on account of an extreme enlargement of the tongue. Dr. H. learnt that at birth his tongue was enlarged and slightly protruded without the maxillary bones, so as materially to interfere with the process of suction. When about a month old, the tongue greatly increased in size, and so far projected from his mouth as to produce considerable deformity; since that period it had grown with his growth.

"When I first examined the case, the tongue projected beyond the upper incisors three inches — circumference six inches—vertical thickness one inch and a half. The tongue exhibited a violet colour; was of a hard and incompressible consistence, covered with a dark slimy secretion, and that portion of it without the mouth was much larger in volume than that within. The weight of the tongue pressing continually on the inferior incisor and canine teeth, gave them a horizontal direction forwards; and at the same time drew the os hyoides and larynx upwards and forwards.

On examining the inferior maxilla, the rami were found much shorter than natural; half an inch at least less in length than that of his brother, who in all other respects was of the same size. The angle of the jaw was unusually obtuse, and the molar teeth were rather longer than natural. By these alterations in the conformation of the parts, the patient had so far overcome the inconvenience arising from his projecting tongue, that the molars could be readily brought in contact, and thus the process of mastication could be performed, and deglutition effected almost as well as if no deformity existed. He was obliged, it is true, to cut his food in small pieces, and introduce it at the side of his mouth." From this the saliva continually dribbled.

At the end of a week, Dr. Harris performed the operation, having previously extracted the inferior incisor teeth.

"I first elevated the tongue, and dissected it from the floor of the mouth, about three-fourths of an inch behind the anterior part of the jaw, and then introduced a strong straight bistoury, commencing where the first incision terminated, and pushed it through the organ between the median line and the left raninal artery, and drew it forward and laterally, so as to form the left flap, terminating at a point corresponding with the teeth. After the divided artery was secured by a ligature, the bistoury was again introduced in a corresponding position on the right side, by which the opposing flap was made; the artery was secured, and then the intervening space was divided by strong scissors. The cut in the tongue resembled in form the letter V inverted. In performing the operation in this manner, I had perfect command of the tongue until after the blood-vessels were tied. The flaps were now approximated, and maintained in this state by means of three interrupted sutures."

A pointed and well-formed tongue was thus constructed. Some topical bleeding was necessary afterwards, but on the fourth day the ligatures could be removed, and on the fourteenth the wound was healed.

From the shortness of the rami of the jaw, and the obtuseness of its angles, the incisor teeth were separated an inch and a quarter, while the molar were in contact. The extraction of some of the latter overcame nearly half of the separation—pressure diminished it still farther—and at the termination of a year the jaws admitted of being nearly closed.

Dr. Harris published a similar case, (save that the tumor was not congenital) in the year 1829, in our contemporary. Baron Percy has collected twelve cases of this disease, from the records of all countries, and commencing with Galen. Of the twelve cases, four were operated on with success—the rest were abandoned to their fate.

* American Journal, May, 1837.

VI. DELIVERY OF A FŒTUS THROUGH THE ABDOMINAL PARIETES. By S. H. HARRIS, M.D., of Clarksville, Virginia.*

Dr. Harris very justly remarks, that many rare and many useful facts are daily lost to science through the "negligence of country practitioners," and, we may add, of town ones too, who dislike the trouble or fear the ordeal of publicity. It were much to be desired that every remarkable circumstance *should* be accurately observed, and *could* be properly recorded. But much has already been effected, and, no doubt, more will be so.

In a late number of this Journal, will be found a case, which was communicated to the Royal Medical Society of Copenhagen, in 1833, by Kjär, district surgeon at Holstebræ, in Jutland. In the case in question, a dead fœtus was extracted piecemeal through an opening which formed below the umbilicus—the opening itself leading to the cavity of the uterus, and not to any accidental intermediate sac.

The case which follows will be found in its most essential respects to resemble the preceding, and, as it must be deemed extremely rare, it deserves, of course, to be accurately recorded.

*Case.* "On the 11th day of November last," says Dr. Harris, "I was called to a negro woman residing in the village, who was labouring under uterine hemorrhage. She was about thirty-five years of age, of robust constitution, and the mother of six living children. According to her own calculation, she was in the eighth month of pregnancy. A few days before, while making up a bed, she had been suddenly seized with pain in the abdomen, which was soon followed by a slight discharge of blood from the uterus. The pain and hemorrhage frequently recurred, but was not so severe as to prevent her from attending to her usual employment, until the evening I was called in. The hemorrhage at this time was somewhat alarming, and the pain very distressing, though confined chiefly to one side of the abdomen. As she was of a plethoric habit, and her pulse full and strong, I bled her freely, and gave two grains of the acetate of lead, with the quarter of a grain of opium, every half hour, and directed the application of cold cloths to the region of the pubis. After continuing this course for six hours, the flooding diminished, but the pain continued without any amendment. I bled her again to the extent, probably, of sixteen ounces, and continued the acetate of lead and opium at longer intervals; some laxative was likewise given, and the cold applications kept applied. Twenty-four hours expired without any material change in her situation, except that the abdomen became more painful to the touch, and the breasts somewhat tumid and tender. The flooding at this time was not profuse, but its continuance induced me to believe that delivery would soon take place. She was again bled twelve ounces, and the acetate of lead and opium discontinued; the cold applications were used only occasionally. After waiting perhaps twenty-four hours, I made an examination of the os uteri, and found it not larger than a twelve and a half cent piece, and very rigid. The finger, instead of passing through a circular opening with thin edges, as at the full period of utero-gestation, passed through a narrow channel of more than an inch in length. It was evident, therefore, that the neck of the womb had not yet expanded. I could distinctly feel the head of the child through the membranes, but the placenta was no where in reach. After thus satisfying myself that it was not a placental presentation, and that the rigid state of the os uteri forbad the administration of ergot, I introduced the tampon, gave fifty drops of laudanum, and left the patient for twenty-four hours. I will mention, by the way, that up to this time and during the progress of the case afterwards, her bowels were evacuated daily by some laxative medicine or mild injections. The opiate procured some sleep, but it had not changed in any respect the general aspect of the case. The os

* American Journal, May, 1837.

uteri remained rigid and unyeilding, and the pain incessant, without appearing to act in the slightest degree on the contents of the uterus. The flooding continued, and though not profuse, its constancy began to weaken the patient, and excite some anxiety in my mind as to its final result. About this time (between three and four days after I commenced treating the case) the tension and soreness of the breast subsided, and she no longer felt the motion of the child. The liquor amnii had likewise been discharged. These evidences of the death of the fœtus were soon confirmed by the offensive character of the discharges. The pain appeared to be fixed at this time in the left hypochondriac region, and remained stationary for many successive days. Her respiration was from the first somewhat obstructed by an accumulation of frothy mucus in the bronchi. This symptom continued throughout, and was a source of great distress and uneasiness."

A particular detail of the symptoms for the next twenty days is not attempted. Various conjectures regarding the nature of the case entered the mind of Dr. Harris; but we need not mention them. About the twelfth day of her confinement, the placenta came away, partially putrid. The stomach rejected the ergot—all prudent efforts to dilate the os uteri were ineffectual—opium produced no more than a temporary respite from pain—nothing, in short availed.

"About the 10th of December, the nurse called my attention to a small circumscribed tumour about the size of a chesnut, a little below the umbilicus to the right of the linea alba. I will remark, that for six or eight days previous to this time, the pain seemed to be confined chiefly to this part of the abdomen. The tumour felt very soft, and communicated to the touch a sense of fluctuation. At its base there could be distinctly traced a muscular ring, very hard, and rather more than half an inch in diameter. Gentle pressure on the tumour did not appear to give much pain; but when the end of the finger was forced into the ring, she complained of excessive pain. The moment I saw and examined this tumour, I felt satisfied that there was a solution of continuity of the uterus, and that the fœtus was making its way to the surface."

As the health of the woman was not materially giving way, Dr. Harris at first applied poultices only, and deferred opening the tumour until the 13th, when it was materially enlarged, while the muscular ring at its base was probably two inches in diameter. A slight incision was made into the apex of the tumour, when about half a pint of thin grayish matter issued, together with a portion of gas, which filled the room with a very offensive odour. A probe was introduced, which soon met with resistance, so as to leave no doubt that the fœtus was fast approaching the surface.

On the 14th, the incision was enlarged, the muscular ring having expanded in the mean time so as to admit the hand. The putrid mass was then drawn to the opening by means of forceps, and a small blunt hook, so as to enable Dr. H. to grasp it with his right hand, and to extract it entire. The bones of the cranium were left behind, and it was necessary to introduce the hand into the body of the uterus, and to bring them away in pieces. The fœtus seemed between seven and eight months old.

Injections into the cavity of the uterus were subsequently resorted to, and the edges of the incision properly brought together. The wound healed in about five weeks, and, while it was doing so, Dr. Harris could at any time distinctly see the lining membrane of the uterus in the bottom of the wound, and could with perfect facility introduce his finger into the cavity of the organ.

### VII. Diseases of the Sandwich Islands.*

An account of these is contained in our contemporary, from the pen of Dr.

* American Journal, May, 1837.

Alonzo Chapin, late a resident missionary at those islands. We shall merely notice a few of the more prominent circumstances.

The *Diet* of the natives is principally vegetable, and consists mainly of the sweet potato and kalo.

The *Climate* is remarkably uniform, and even pleasant, although the islands are situated between 18° 50′, and 22° 20′ North latitude, and 154° 53′, and 160° 15′ West longitude.

The *Diseases* most common, within Dr. Chapin's observations, were *fevers, opthalmia, catarrhs and asthma, rheumatism, venereal diarrhœa,* dysentery, *cutaneous diseases, scrofula, dropsy,* &c., and they occurred, in frequency, in about the order in which they are set down. Diseases sometimes occur epidemically, as was the case with catarrh repeatedly, and croup once during his residence at the islands. Many other diseases, not specified, were often met with.

The *Fevers* are not very fatal, and, when idiopathic, usually remittent.

*Opthalmia,* of the purulent form, abounds in every portion of the group, and opaque corneas and thickened coats of the eyes, are very numerous. The old and the young are alike affected by the disease; very small children are occasionally met with nearly blind from its effects.

*Asthmas* and *Catarrhs* are frequent, but the latter are not severe. The bad clothing, and as bad habitations of the natives, are calculated to favour the occurrence of such diseases as arise from the operation of cold. Thus *Rheumatism* is very common.

*Venereal Disease.* If it be a fact, says Dr. Chapin, that the aborigines of America were affected by syphilis and gonorrhœa before Europeans visited them, or if, as is presumed by Dr. Thompson, "syphilis has been thousands of times generated *de novo* by impure sexual intercourse," it is certain that neither disease existed, or was known at the Sandwich Islands before the visit of Captain Cooke in 1779.

"With such an introduction, the venereal disease has for the past fifty-seven years continued to spread and increase; perpetuated and extended too by almost every vessel which touches at the islands, till words would fail to express the wretchedness and woe which have been the result. Foul ulcers, of many years standing, both indolent and phagedenic, every where abound, and visages horribly deformed—eyes rendered blind—noses entirely destroyed—mouths monstrously drawn aside from their natural position, ulcerating palates, and almost useless arms and legs, mark most clearly the state and progress of the disease among that injured and helpless people."

Dr. Chapin observes that the natives possess, of themselves, no means of controlling the disease, and but few of them have ready access to foreign physicians. Most of them permit secondary symptoms to appear before they seek assistance. Is the picture favourable to the non-mercurial treatment? We apprehend not.

*Diarrhœa and Dysentery.* "These have, besides the usual exciting causes which prevail in most places, an additional fruitful source, in a blind and barbarous practice of using immoderately the most powerful and drastic cathartics. The inside of the calabash (*Cucurbita lagenaria,*) triturated seeds of the castor oil, the fruit of the candle nut, (*Aleurites triloba,*) two or three species of *Ipomeæ* and some other drastic articles are given in such doses as sometimes to create the most obstinate and dangerous dysenteries. I have known a case in which the average operations of four cathartics, given to disperse dropsy, were twenty-one, the aggregate eighty-four, and another case in which a man from a fear that he would be sick, took such an enormous dose of the calabash as to produce a hemorrhagy which proved fatal within a few hours."

*Cutaneous Diseases and Scrofula* are common from their filthy habits, imperfect clothing, and indifferent food.

*Hepatitis* is rare.

*Diseases of Females.* The females mostly marry before the age of puberty, and but few pass the first catamenial period without having had sexual intercourse; and the opinion is prevalent that the menses are the effect of coition, and their appearance in an unmarried girl is regarded as evidence of illicit conduct. This universal practice of premature sexual intercourse, is probably the principal cause of unfruitfulness among the women. How far they are affected by the venereal disease, is not certain. Multitudes live childless, and cases are rare of a woman's giving birth to more than four or five children.

*Fluor albus* is universal, and exists abundantly in its worst forms; uterine hemorrhage is frequent and obstinate. *Displacements of the womb* are of common occurrence.

*In parturition the women* are not specially favoured, but suffer *equally with the labouring class of females elsewhere.* Some of Dr. Chapin's severest cases of midwifery have been among the islanders. In several instances, the uterine and vaginal rigidity has been excessive.

Of course, the natives have no knowledge of *medicine.* Charms and incantations are prominent remedies. Many have been pounded and roasted to death, from a belief that their diseases were the effect of an indwelling spirit.

*Population.* This has enormously decreased since Capt. Cooke's visit in 1779. Then the numbers of the islanders were estimated at 400,000—now they only amount to 135,000. One of the missionaries computes that there are annually 6838 deaths and 3335 births. If so, the native inhabitants will at no great distance of time disappear. The main causes of the decrease are—the venereal disease—and the abuse of alcoholic liquors. It is, then, with the Sandwich islanders, as with the Aborigines of America, the white man's arts and vices have corrupted and destroyed them. A melancholy reflection.

### Case of Worms in the Urinary Bladder, simulating Stone in that Organ.*

The patient was a lady, aged 35, living at Hartland. She had most of the symptoms of calculus in the bladder, and was several times sounded by her ordinary physician, as well as by Dr. Brigham, who relates the case.

This latter gentleman was confident that he felt some unnatural substance or tumor in the bladder, but could not determine its nature or extent. One day, however, the lady voided from that organ, with considerable pain, a round white worm, and from that moment her complaints ceased.

It subsequently turned out, that, when she was fourteen years of age, she was troubled with difficulty of making water, and discharged a worm an inch long from the bladder—and, in six weeks after that, she passed another of the same size, when her complaints ceased.

Dr. Brigham refers to a case related by Mr. Lawrence in the Medico-Chirurgical Transactions.† The patient in that case passed several hundreds of worms, some quite small, some from four to six inches in length.

---

### Preparation of Phloridzine.‡

Place in a copper vessel the bark of roots of apple trees, freshly dug up, and pour in enough of distilled water to cover the bark entirely. Boil for four hours, always replacing the water which escapes by evaporation. After four hours, pour off the liquid, and then add a fresh quantity of water to the residue equal to the first, and boil it again, but only for one hour, and then pour off. The two products, when kept at rest for thirty hours, will deposit a great quantity of phloridzine, re-

---

* American Journal, May, 1837.

† Vol. II.

‡ Journal de Chimie Médicale. Med. Gazette.

sembling dark velvet. Collect the phloridzine, and add to it an equal quantity of animal carbon: boil the two in a little water, and filter. This process must be repeated three times, and when the liquid is made to cool gradually, a beautiful crystallization of the phloridzine takes place. M. B. has been unable to discover this substance in the bark of the root of cherry trees.

Phloridzine is very light, of a white colour, with a slightly bitter, but not at all astringent taste. It gives no reaction with test-paper. It is scarcely at all soluble in cold water, but much more so in hot alcohol, which at its common temperature dissolves half its own weight of it. Being treated with half its weight of nitric acid, to which water was added, after an hour of boiling the liquid assumed a red colour, and no traces of phloridzine could any longer be detected.

---

### OPINIONS OF NAPOLEON ON MEDICAL EDUCATION.

The following passage from Basil Hall's "Napoleon in Council," displays the opinions, we should rather say fancies, of that wonderful man, on the subject of Medical Education.

"With respect to the degrees given by the University, that of Doctor ought not to be too readily bestowed. The candidates ought to be examined on the most difficult subjects—for example, on the comparison of languages; and it would not be amiss were they required to converse in Latin for an hour and a half. It is by no means necessary that all the world should be rendered eligible for a Doctor's degree; nor do I approve of the condition which requires that a Bachelor of medicine should first take a scientific degree; for medicine is not a positive and exact science, but one of observation and conjectures. For my part, I should have more confidence in a Doctor who had not studied the exact sciences than in one well acquainted with them. I preferred M. Corvisart to M. Hallé, because M. Hallé belongs to the Institute, whereas M. Corvisart does not know what is meant by two triangles being equal to one another! The student of medicine ought not to be disturbed in his visits to the hospital or the dissecting-room, or in his medical studies. Anatomy, though the least uncertain branch of the art, is still enveloped in darkness. We know neither why we live, nor how we live, nor what the living principle is. To require, therefore, that a young man shall be versed in knowledge of such different kinds before he can enter upon his profession, is to risk losing the public services of the great men whom such a profession might turn out; for by a strange caprice in the structure of the human mind, it may well happen that a man may be a great physician, or a great jurist, who could never work a sum in compound division."

It would be throwing away our time to point out the many fallacies contained in these whimsical opinions. But fallacies are not Napoleon's alone—they are frequently expressed in society. Because medicine is not an exact science, *therefore* a medical man should not have an *exact* habit of thinking. Why the object is to make medicine as exact as possible, and this is most likely to be effected by a man who *is* accustomed to exact reasoning. The best proof of the correctness of this idea, is the improvement which has actually been worked in medicine by the cultivation of healthy and morbid anatomy—in other words by the cultivation of those means which tend to further its exactness.

It is absurd to urge against anatomy that we know nothing of the intimate essence of life. It would be just as rational to urge against physics that we know nothing of the nature of attraction. But we can learn much of the laws both of life and of physics, and this is a sort of knowledge which is highly useful to us. Half a loaf is surely better than no bread.

A classical education is no doubt of service in the making of a medical man. But so is all education, and, so far as immediate utility goes, the classical is inferior to the mathematic. The great advantage of classical learning is its peculiar aptitude for refining the taste, and for opening many sources of intellectual enjoyment and improvement. A physician is to mingle with the higher classes of society, and he must possess something of the manner both of acting and of thinking, which distinguishes those classes. Their minds, from the character of public schools and universities, are deeply imbued with classical recollections, and a man who has them not cannot fail to disgust.

---

### Report on the National Vaccine Establishment.*

The following short extracts from this Report are gratifying, so far as they go.

The annual loss of life by small-pox in the metropolis, and within the bills of mortality only, before vaccination was established, exceeded 5,000; whereas in the course of last year only 300 died of that distemper; and it is probable that even this mortality, however comparatively small, is owing to the continued partial practice of inoculation, which is liable to disseminate far and wide its contagious influence, to the imminent danger of all who have not been protected by previous vaccination, or by having had the disease already.

Although we lament sincerely the mistaken judgment which prefers inoculation to vaccination, whether on the supposition, amongst other ill-founded notions of which we sometimes hear, that the original virtue of the vaccine virus has been worn out by time, or on any other equally ill-grounded opinion, we have the satisfaction of knowing that vaccination has made considerable progress since our last report, and that we have supplied lymph, not only to every part of this kingdom in the course of the last year, but to all the colonies also, and to many of the capitals of Europe.

* Med. Gaz., July 22.

### Vicarious Menstruation from the Skin of the Thorax.*

Dr. Cowan, physician to the Reading Dispensary, relates this case. It is a curious one.

*Case.* Mrs.B., aged 49, of industrious habits, and robust health, mother of five children, and never liable to uterine irregularity or leucorrhœa, about five years since, in consequence of fright during the menstrual period, experienced suddenly a suppression of the catamenia, which have never since returned. Her general health was not sensibly affected, but two months afterwards a sudden and copious discharge of blood, per anum, took place, from which she suffered no inconvenience. Two or three months later she was sensible for the first time of a pricking sensation on the under surface of the left mamma, which was soon followed by increased heat and redness, and a discharge of a thin serous, colourless fluid, similar in no other respect than as regards the smell, to the natural menstrual secretion. This continued for about twenty-four hours, when the surface gradually dried up, desquamation followed, and the skin in a few days resumed its natural appearance. With the cessation of the discharge of the breast first affected, a precisely similar series of phenomena took place in the corresponding point of the opposite side, and has continued up to the present moment, an interval of more than four years, to be repeated at the regular monthly period. She only complains of the local smarting and the disagreeable smell, which at times she states to be almost insupportable.

The affected part occupies a space about the size of the palm of the hand, and is situated in the fold of the skin between the breast and the thorax, extending equally upon both surfaces. There is an exact correspondence on either side, and during the period of secretion the skin has all the appearance of a raw and blistered surface;

* Med. Gaz., August 5.

the nipple is tender, but has never been subject to any discharge.

She says that the local changes have not sensibly varied in character or extent from the commencement.

---

SUBSTITUTE FOR THE "LIQUOR OPII SEDATIVUS" OF BATTLEY.*

Mr. Houlton, of Lisson Grove, speaks very highly of the following preparation.

"Take two ounces and a half of the best Turkey opium; thirty-two fluid ounces of Beaufoy's acid, the strength of pickling vinegar: macerate with a gentle heat for six days, frequently shaking the vessel; then filter, and evaporate the fluid to the consistence of the extracts of the Pharmacopœia, finishing the evaporation by the spontaneous method. (*This I employ under the name of Extractum Opii Aceticum.*)

Take the above extract; alcohol, five fluid ounces; distilled water, thirty-five fluid ounces. Macerate for eight days, and filter.

This liquor opii will be about of the strength of tinctura opii in sedative property; but the strength may be varied at the will of the apothecary. I prefer it of the proportions I have stated."

---

ON POISONING WITH THE ESCULENT MUSHROOM. By D. O. EDWARDS, Esq.

Mr. Edwards relates the following cases, which we must abridge, in the Lancet.†

F. B., aged 25, Anne B., his wife, aged 23, and their child, aged 4, gathered a large quantity of mushrooms in the morning of the 20th of August, 1830, and not being able to dispose of them, went home, cooked them all, and made a meal of them, taking nothing besides but water.

In half an hour they were affected with giddiness, and the subsequent symptoms may be thus traced in the man. The giddiness gradually increased until a dimness of sight supervened. He then appeared to himself as if involved in flame; the hearing became painfully acute, and objects (probably from the rapidity of the sensations) became confused to the eye. He occasionally felt a sentiment of uncontrollable gladness, which prompted him to the muscular movements. Yet he was fully conscious that he was in a state of preternatural excitement. The woman gave a recital of her sensations similar to that of the husband; but the condition of the child could only be gathered from the obviously excited irritability.

It was about an hour after the meal when Mr. Edwards was summoned to see them in the waiting-room of the Westminster Hospital. He thought at first they were all drunk. The man, woman, and child, were in continual motion, either dancing or throwing themselves into grotesque attitudes. Their countenances expressed the highest hilarity, and their consciousness was quite unclouded. On being charged with drunkenness, the adults exhibited the most lively indignation. The man was most vividly affected by the poison; his eyes glistened; the pupils expanded; the pulse was full and frequent; no sordes could be detected on the lips or teeth; the tongue was clean, and the breath untainted. He conversed without embarrassment, and said that he was intelligent of every thing that passed around him.

Mr. Edwards used the sulphate of zinc, as an emetic, but without benefit. The man, indeed, began to exhibit symptoms of congestion of the brain. His stomach was washed out with Reed's pump, and some half-digested mushrooms were in this way removed. The man was then quickly relieved, and a few leeches being placed upon the temples, he was discharged well, with his family, next day.

---

* Med. Gaz., August 12.

† July 1, 1837.

### Cure of Club-Foot by the Division of the Tendo Achillis.*

The case is related, with all the delight of a grateful father, by Mr. Ray, a highly respectable surgeon, of Milton, in Kent.

The case was that of his eldest son, a healthy boy, born in Feb. 1823, with a club-foot. This was not considered very bad; yet all mechanical means failed to remedy it. The state of the boy before the performance of the operation was as follows.

In the erect posture the patient appears to be simply standing upon the toes and ball of the foot of the affected limb, the heel being drawn up by the muscles of the calf as high as possible, not touching the ground by three inches; in consequence of the extreme rigidity of the tendo-achillis and gastrocnemii muscles, it is impossible to place the foot in its natural position by bringing down the heel. In walking the patient limps a good deal, and treads on a very small part of the sole; there is not much inclination of the foot inwards, though he treads more upon the ball of the little toe than of the great toe. In the act of walking there is no bending at the ankle-joint. Although the entire affected extremity is rendered longer, through the extension of the foot, the leg is, in reality, somewhat shorter than that of the opposite side. The tibia and fibula are also more slender; the inner malleolus presents itself more anteriorly, and the other more posteriorly, than natural. The heel being drawn up as much as possible, causes the foot to appear to be a continuation of the leg; it is, in fact, nearly in a straight line. The arch of the foot is much greater than natural; the tarsal bones, therefore, present a great projection upwards; the affected foot is an inch and a half shorter than its fellow; the muscles of the leg are much less developed than those of the sound limb; there is no paralysis of the tibialis anticus, peronei, or other muscles on the front of the leg; he can put them in action at pleasure, without, however, being able to move the foot much.

On the 1st of May, the operation was performed in London by Dr. Little. This gentleman divided the tendo-achillis, about an inch and a half above the malleolus, by introducing a very narrow, slightly-curved, bistoury between the tendon and the deeper-seated muscles and tibial vessels, directing the edge of the knife against the front of the tendon, dividing it from within outwards, leaving the skin covering the tendon untouched. The section was accomplished most skilfully, and the *minute* puncture in the skin was covered with a strip of adhesive plaster, and a loose bandage was applied round the foot and leg. Any separation of the divided ends of the tendon was prevented by placing the foot in its deformed position upon a stiff pasteboard splint, applied along the outer side, and by adjusting the limb, with the knee bent, upon a pillow.

On the 4th day, the puncture was firmly cicatrized, and Stromeyer's foot-board was applied. By the 14th day, the lymph between the ends of the divided tendon was sufficiently stretched to allow the foot to be brought to a right angle with the leg.

19th day. To-day the use of Stromeyer's foot-board is partly discontinued, and he wears in its stead Scarpa's shoe, modified by Stromeyer, which tends better to overcome the too great curvature of the bones of the tarsus, now that the foot is bent to the right angle. He walks about in it very well; the entire sole and the heel touch the ground; nor does the foot, when removed from all apparatus, return to its vicious position. The arched form of the dorsum of the foot, and the inclination of the front row of the tarsal and metatarsal bones, inwards, is now more striking to the casual observer than before.

At the end of six weeks and a few days, the boy returned home, and has since been steadily improving.

This must be looked on as a very valuable addition to our stock of operations.

* Lancet, July 15, 1837.

### Spontaneous Cure of a Gelatinous Polypus Nasi.*

A woman, aged 30, while labouring under an attack of hepatitis, became affected with a gelatinous fungus in the left nostril. It grew from a small peduncle, and took its origin from the middle meatus of the nose. The patient obstinately refused to allow Mr. Maddock, who relates the case, to remove it. It still grew—filled the nose—extended backwards to the fauces, forwards out of the nostril—was accompanied with headaches—and was the source of a constant fœtid discharge. Matters seemed going on very badly, but gradually the fungus decreased, the discharge being mixed with drops of blood and small portions of fungus—the headache passed away—and at last the fungus disappeared altogether.

If there is no return of it, the patient may consider herself lucky.

---

### Opinions on the Contagion of Glanders.†

"Glanders, the nature of which is yet a problem, is it contagious? Huzard says, 'yes,' with Solleysel, La Guirinië, and Gaspard Saunier. Camper, Godine, Magendie, Dupuy, say, 'no.' La Fosse says, 'yes' and 'no.' Coleman, Delabere Blaine, and Dutz, neither say yes nor no! Chabert, after having, during his whole life, maintained the contagiousness of glanders, has retracted this opinion in his old age."

A very pretty specimen of the certainties of veterinary as well as other medicine.

---

### Dr. Graves' Opinion of the Pathology of Phlegmasia Dolens.

Our readers are well aware that the following doctrine has been advocated in this Journal for 20 years past.

"Let me now direct your attention to the case of Rebecca Howard, who came into hospital on the first of this month, eight days after her accouchement, with painful swelling of both lower extremities. From the history of her case it appears, that three or four days after her confinement, she got severe pain about the heel and inner ankle, accompanied by swelling, which, commencing about the same situation, extended rapidly up the thigh as far as the groin. A similar swelling appeared likewise in the other limb, but instead of commencing below, it appeared first in the upper third of the thigh, and afterwards spread downwards, attended with violent pain, apparently in the course of the great sciatic nerve. Along the course of the veins a number of hard cords, extremely tender to the touch, could be distinctly felt; the lymphatics, though somewhat tender also, did not seem to be so much engaged, and there was no inflammation of the glands of the groin.

Here we had a case of phlegmasia dolens, or in other words, painful inflammatory œdema of the lower extremities, involving the skin, subcutaneous cellular tissue, veins, and lymphatics, more or léss distinctly. I have before stated to you my opinion, that this affection does not necessarily depend on phlebitis; on the contrary, I think that in the majority of cases the disease commences in the subcutaneous cellular tissue, and afterwards extends to the veins and lymphatics. Observe the course of the inflammation in both limbs. In one it commences in the vicinity of the inner ankle, and extends up the thigh; in the other it is first observed in the upper part of the thigh, and spreads downwards. Now, where œdema is the consequence of phlebitis, or where it is artificially produced by tying or compressing one of the large venous trunks, it is always first observed in the lower part of the limb. You perceive, then, that those who explain the occurrence of phlegmasia dolens by referring it exclusively to phlebitis, are not able to account for it as commencing in the thigh and spreading downwards. But how much easier is the

* Lancet, July 15, 1837.

† Lancet, August 5, 1837.

explanation, if we look upon it as a peculiar inflammation of the subcutaneous cellular membrane of the limb, involving in its progress to a greater or lesser extent, the veins and lymphatics, and sometimes extending to the joints. From this view of the pathology of phlegmasia dolens, you can understand why the upper part of the thigh may become primarily affected, and that effusion may take place above before it occurs below.

So far with respect to the pathology of the diseases: now with regard to treatment. In attempting to remove this inflammation, we were obliged to keep clear of any measures calculated to increase constitutional debility. The woman, though young, was of a delicate constitution; and there is this peculiar difficulty in the treatment of diseases after parturition, that they occur at a time when the patient has been more or less debilitated by the efforts of labour and its consequences. Our object, therefore, was to reduce the local inflammation, at the same time that we endeavoured to support the woman's strength by a light and nutritious, but not heating, diet. We commenced with the application of leeches, to the number of ten, along the inside of each limb; these we repeated to the same amount on the following day. In the application of leeches in cases of this kind, you must be guided by the circumstances of pain, tension, and swelling; these are sometimes greater in one portion of the limb than in another, most frequently in the course of the veins; but you should always take care to have them applied over those spots in which the inflammatory process seems to exist in greatest intensity. Our next step was to open her bowels by means of purgative injections, to be repeated as occasion required. In addition to this, I directed the limb to be gently rubbed with an ointment composed of one ounce of mercurial ointment, two ounces of lard, and three drachms of extract of belladonna. I have already dwelt so often on the local, antiphlogistic, and narcotic effects of this composition, that it is unnecessary for me to say any thing of it at present.

With respect to internal remedies, I ordered her to take five grains of Plummer's pill every night and morning; but as this produced griping and a tendency to diarrhœa, we were obliged to change it for hydrarg. c. cretâ, with Dover's powder. On the 24th (the fifth day of her treatment) her mouth became affected, and the pain along the sciatic nerve, as well as the general soreness of both extremities, decreased. I forgot to observe, that from the commencement we had given opiates freely; indeed, this was one of the principal parts of our treatment. She first took the liquor of the muriate of morphia, in doses of twenty drops, three times a day; this we exchanged for opiate injections, when her bowels became irritable under the use of Plummer's pill. On the 24th there was a considerable improvement in her symptoms, as I have already stated; but she was very weak; there was still considerable soreness of the extremities, and she complained of pain and tenderness in the right groin, shewing that the lymphatics as well as the veins were engaged. I ordered the opiate enema to be repeated, and allowed her the free use of chicken-broth, rice, and a small quantity of wine. On the 25th she was directed to take a pill containing half a grain of opium every third hour. Next day the report states that she finds herself much better, that her bowels are quite natural, that she feels no pain in the lower extremities, except when pressed or moved, and that she has regained the power of her limbs. Two days afterwards she was able to stand, and at present she is so far recovered that I intend to dismiss her to-morrow.

The treatment of cases of this description involves some very curious and important considerations. With the exception of leeching, the treatment which we employed in this case cannot be called antiphlogistic; for throughout the whole course of the disease we gave opium freely, allowed her a nutritious diet, and after the first four or five days the use of wine. This shews that, in diseases called inflammatory, no general rule of treatment can be laid down, and that our practice must vary in the most remarkable manner, according to circumstances. Had I treated this inflammation by leeching, low diet, purgatives, and antimonials, it is very probable she would have sunk. But while we were endeavouring to subdue local inflammation by leeching and mercurial ointment, we supported the constitution by a proper diet, nourishing but not heating, and afterwards by the use of wine. At the same time we gave opium in free and repeated doses, with the view of diminishing pain and irritation, and procuring sleep—a most important matter in the treatment of all acute affections combined with irritability. We also gave mercury internally, because it has been found extremely valuable in such cases, when given rather as an alterative than with the view of rapidly and violently affecting the system. Under this plan of treatment her convalescence has been very rapid. It is a plan abundantly simple, but one which I can recommend to you with confidence.

With respect to the after-treatment of this case, I have merely to observe, that as soon as the hyper-sensibility of the limbs became diminished, I ordered them to be rubbed diligently twice a day with warm olive oil. How this acts I cannot distinctly say; but it appears to diminish tension, to promote absorption, and to increase the pliability of the limbs. Latterly we have given up this, and had recourse to dry friction and bandages. At present she is taking, three times a day, a mild tonic draught, composed of tincture of orange peel, half a drachm; tincture of hops, twenty minims; carbonate of soda, five grains; water, an ounce."*

* Med. Gazette, 19th Aug. 1837.

